# Gestational diabetes mellitus–induced adipokine dysregulation and links to metabolic programming risks

**DOI:** 10.3389/fendo.2026.1776904

**Published:** 2026-05-12

**Authors:** Jolanta Lis‐Kuberka, Marta Berghausen‐Mazur

**Affiliations:** 1 Department of Biochemistry and Immunochemistry, Division of Chemistry and Immunochemistry, Wroclaw Medical University, Wroclaw, Poland; 2 Department of Neonatology, J. Gromkowski Provincial Specialist Hospital, Wrocław, Poland; 3 Faculty of Medicine, Wroclaw University of Science and Technology, Wroclaw, Poland

**Keywords:** adiponectin, birth weight, free leptin index, gestational diabetes mellitus, leptin, leptin-to-adiponectin ratio, maternal and cord plasma, maternal–fetal axis

## Abstract

**Introduction:**

Gestational diabetes mellitus (GDM) is a pregnancy−related hyperglycemic disorder with highly variable global prevalence, largely driven by differences in diagnostic criteria, maternal characteristics, and lifestyle factors, which complicates efforts to standardize screening and prevention. GDM disrupts maternal–fetal glucose homeostasis, leading to fetal exposure to hyperglycemia and hyperinsulinemia, and is associated not only with immediate obstetric complications but also with long−term metabolic risk in both mothers and offspring.

**Objective:**

This study evaluated the impact of maternal hyperglycemia, classified as diet−controlled (GDM−diet) or insulin−treated (GDM-insulin), on leptin, adiponectin, soluble leptin receptor (sLeptinR), and the derived indices leptin−to−adiponectin ratio (LAR) and free leptin index (FLI) across the maternal–fetal axis.

**Design:**

This study was a retrospective observational cohort study targeting patients with gestational diabetes mellitus. Maternal and cord blood plasma from 30 hyperglycemic and 23 normoglycemic mothers were analyzed for leptin, adiponectin, and soluble leptin receptor concentrations using immunoenzymatic assays.

**Results:**

Maternal plasma leptin concentrations were significantly higher in the GDM-insulin group (10.05 ng/mL) than in non-GDM pregnancies (4.44 ng/mL; *p* = 0.033). The leptin-to-adiponectin ratio was also elevated in GDM-insulin (4.99) compared to GDM-diet (2.18; *p* = 0.043). No significant between-group differences were observed for adiponectin, soluble leptin receptor, or the free leptin index. In cord blood, adiponectin concentrations were higher in GDM-diet neonates (9.05 μg/mL) than in GDM-insulin (5.70 μg/mL; *p* = 0.010). Maternal leptin and its derived indices (LAR, FLI) were associated with maternal overweight/obesity, whereas maternal adiponectin and cord blood leptin were related to neonatal birth weight.

**Conclusion:**

These findings point to leptin, adiponectin, FLI, and LAR as possible metabolic indicators of GDM-linked insulin resistance, and low-grade inflammation. Higher maternal leptin, LAR, and FLI, alongside stable sLeptinR, signal leptin resistance, which disrupts placental nutrient transport and links maternal metabolic stress to fetal anthropometric measures. Tracking these markers could guide postnatal steps to cut long-term metabolic risks across the next generations.

## 1 Introduction

Gestational diabetes mellitus (GDM) is a form of hyperglycemia diagnosed during pregnancy, and this state is a significant risk for both the mother and her fetus. The prevalence of GDM varies widely across regions, ranging from ~7.8% in Europe to nearly 28% in the Middle East and North Africa (1–5). This variation reflects differences in diagnostic criteria, genetic susceptibility, maternal age, lifestyle patterns, and obesity prevalence (6, 7). Standardization of screening and prevention remains a major challenge in managing this global health issue.

The biological mechanisms driving GDM remain only partially understood (8–10). GDM is associated with hormonal, anatomical, and metabolic adaptations to pregnancy that disrupt glucose regulation. A pivotal factor in the development of GDM is inflammation, which promotes insulin resistance. Pregnancy itself entails a 30%–50% increase in adipose tissue, amplifying adipokine secretion and macrophage infiltration, thereby generating a state of low−grade inflammation (11–14). The observed systemic pro-inflammatory state in GDM is characterized by elevated interleukin-6 and tumor necrosis factor-α, which impair insulin signaling and contribute to hyperglycemia (15–17). In addition, hyperglycemia may be linked to aberrant maternal immune adaptations to the developing fetus, leading to overexpression of pro-inflammatory mediators (18–20). Pregnancy is normally characterized by cyclical shifts in immune function: a pro-inflammatory Th1-type response predominates in the first trimester (implantation) and third trimester (parturition), while a Th2-type anti-inflammatory profile dominates in the second trimester to maintain tolerance to the developing fetus (20–24). Disruption of this tightly regulated balance is thought to impair placental adaptation and ultimately glucose metabolism. Immune maladaptation, in conjunction with maternal excessive weight gain, exacerbates inflammation, insulin resistance, and vascular dysfunction, thereby predisposing to pregnancy complications such as GDM and to abnormal fetal growth (20).

According to the developmental origins of health and disease hypothesis, adverse intrauterine conditions predispose offspring to long-term metabolic and cardiovascular disorders by disrupting fetal growth and developmental programming (25). Consistent with this view, GDM is associated not only with short-term complications such as preeclampsia, preterm delivery, macrosomia, or growth restriction (26–28) but also with long-term adverse outcomes: children exposed to GDM *in utero* are more likely to develop obesity and type 2 diabetes (29, 30), while mothers face elevated risks of cardiovascular disease, kidney dysfunction, and certain cancers (31–34).

Leptin and adiponectin mediate inflammatory and metabolic dysregulation in gestational diabetes mellitus (35–40). Leptin exerts pro-inflammatory and immunomodulatory effects (41, 42), whereas adiponectin promotes insulin sensitization and anti-inflammatory actions (43). Maternal leptin increases from the first trimester (normal BMI: 13.5 ng/mL; overweight/obese: 30 ng/mL) to the second (normal BMI: 20.1 ng/mL; overweight/obese: 36.6 ng/mL) (44, 45), plateaus or slightly declines in the third (normal BMI: 20.1 ng/mL; overweight/obese: 33.1 ng/mL), and drops postpartum to 10–14 ng/mL—resembling non-pregnant levels (7–15 ng/mL) (46, 47). Maternal leptin is associated with total fat mass, food intake, and energy expenditure (35, 38). Conversely, maternal adiponectin decreases across pregnancy, alongside rising insulin resistance and gestational weight gain, from highest levels in the first trimester (13.3 ± 3.6 μg/mL) to lowest in the third (11.2 ± 3.7 μg/mL) and low levels in maternal circulation might predict GDM and adverse maternal-fetal outcomes (48). Adiponectin concentrations in women's circulation—particularly among those who suffered GDM—usually increase within 3–12 months postpartum (49).

The leptin-to-adiponectin ratio (LAR) is considered a useful index of adipose tissue dysfunction (50–54). In healthy individuals, the LAR is typically 1:2, while an elevated LAR reflects an imbalance between pro- and anti-inflammatory adipokines, linking it to endothelial dysfunction, systemic inflammation, insulin resistance, and heightened risks for type 2 diabetes and cardiovascular disease (11, 13, 43, 50, 52, 55–57). It was reported that LAR has potential as an early biomarker for identifying at-risk individuals of GDM, allowing better preventive care (54). In GDM, these dynamics are altered: studies report reduced adiponectin, elevated leptin, and an increased LAR (36, 39, 58–61).

The bioactivity of leptin is strongly influenced by the soluble leptin receptor (sLeptinR), which binds leptin and regulates the amount of free (bioactive) hormone (38). Maternal soluble leptin receptor in plasma typically ranges from approximately 20 to 30 ng/mL during pregnancy (62), decreases with advancing gestation, and rebounds post-delivery approximately day 2 postpartum (63). Maternal sLeptinR concentrations inversely correlate with leptin and weight gain, reflecting a compensatory mechanism restraining leptin exposure when secretion is high (62, 64). These changes modulate the free leptin index (FLI), defined as the leptin/sLeptinR ratio, a well-established marker of leptin bioactivity and leptin resistance. Elevated FLI, combined with reduced sLeptinR, has been implicated in obesity, type 2 diabetes, metabolic-associated fatty liver disease, and reproductive disorders (65–67). In pregnancy, FLI alterations may contribute to maternal leptin resistance, sympathetic overactivity, and hypertensive phenotypes, positioning it as a potential biomarker of preeclampsia and possibly GDM (68).

Recent evidence highlights alterations in maternal and fetal leptin and adiponectin profiles during pregnancy. However, comprehensive evaluations of adipokines—including leptin, adiponectin, LAR, sLeptinR, and FLI, remain limited, particularly in GDM and its management. We hypothesize that maternal exposure to GDM, stratified by diet-controlled (GDM-diet) versus insulin-treated (GDM-insulin) management, dysregulates leptin and adiponectin levels, as well as leptin-related indices such as LAR and FLI, across maternal circulation with potential translation into cord blood plasma. In this study, we assessed GDM's on adipokine profiles across the maternal–fetal axis and examines associations between maternal BMI, neonatal anthropometry, and adipokine concentrations to inform fetal growth assessment and risk stratification for metabolic complications.

## 2 Materials and methods

### 2.1 Study design

This retrospective observational cohort study included women with gestational diabetes who delivered at the First Department and Clinic of Gynecology and Obstetrics, Wroclaw Medical University (Wrocław, Poland). The issues with sampling collection, especially cord blood (e.g., inappropriate test tube, medical complications during delivery, hemolysis of sample) reduced the final cohort to 53 mother–newborn pairs, resulting in a 7.2% sample size bias [calculated using Calculator.net (https://www.calculator.net/), assuming 95% confidence level (*α* = 0.05) and population proportion: 7.8%].

### 2.2 Recruitment of mothers

For the study, women with GDM (hyperglycemic group; GDM group) and normoglycemic mothers (non-GDM group), were recruited. The selection criteria of women with GDM were an abnormal fasting blood glucose level and/or oral glucose tolerance test (OGTT) following ingestion of 75 g of glucose between 24 and 28 weeks of gestation (69–73), and aged <45 years..

The health status of the mothers and newborns was noted and included the following variables: women’s race, age, preconception BMI, gestational age, type of delivery, newborn’s birth weight, and newborn’s gender. The exclusion criteria enrolled multiple pregnancies, chromosomal abnormalities, preterm birth (<35 weeks), uncontrolled thyroid disease, epilepsy, alcohol consumption, or smoking during pregnancy. Overall, 53 women were enrolled for the study (GDM *n*=30 and non-GDM: *n*=23). Additionally, mothers with pregnancy hyperglycemia were divided into two groups: diet-controlled (GDM-diet, *n*=16) or insulin-treated (GDM-insulin, *n*=14).

### 2.3 Ethics

The Ethics Committee at Wrocław Medical University approved the study (approval code: KB-200/2023N; approval date: 31st October 2023), and informed written consent was obtained from all the mothers enrolled for the research. The study complied with the Good Clinical Practice and Declaration of Helsinki.

### 2.4 Plasma and cord blood plasma collection

The maternal blood and cord blood samples were collected from women at the First Department and Clinic of Gynaecology and Obstetrics, Wroclaw Medical University. Maternal blood samples were collected from puerperas on the second postpartum day, while cord blood samples were collected immediately after vaginal delivery/cesarean section from the umbilical artery. All blood samples were collected into tubes containing 3.2% sodium citrate as an anticoagulant. Plasma samples were obtained by centrifugation for 10 min at 2,000×*g*, aliquoted, and stored at −78°C until analysis (74, 75).

### 2.5 Maternal and perinatal characteristics

Maternal characteristics were assessed using a standardized questionnaire and included age at delivery, parity, race, smoking status, and the occurrence of pregnancy complications (preeclampsia, eclampsia, or chronic hypertension). Pre-pregnancy body mass index (BMI) was calculated as weight (kg) divided by height squared (m²). Mothers were categorized into two groups according to BMI: women with BMI ≥25 kg/m² were classified as the overweight/obese (OWO) group, and those with BMI <25 kg/m² as the normal-weight group.

Perinatal data were extracted from medical records and included infant sex, mode of delivery, growth status at birth, gestational age (GA), birth weight, and the occurrence of GDM. GDM was diagnosed between 24 and 28 weeks of gestation using the OGTT.

### 2.6 Birth weight relative to gestational age

Neonatal birth weight was categorized as follows: small for gestational age (SGA; <10th percentile), appropriate for gestational age (AGA; 10th–90th percentile), or large for gestational age (LGA; >90th percentile) (76).

### 2.7 Determination of leptin and adiponectin concentrations

The levels of leptin and adiponectin were measured using a commercial ELISA kit (Biorbyt, Cambridge, United Kingdom). The detection ranges of the enzyme-linked immunosorbent assays for both adipokines were 62.5–4,000 pg/mL. The sensitivities of the tests were <10 and 7 pg/mL, respectively.

All plasma samples were assayed in duplicate. Plasma samples were diluted (10 times for leptin and 10,000 times for adiponectin). Intra- and interassay coefficients of variation were as follows: for maternal leptin, 4.5% and 9.2%; for cord leptin, 1.0% and 4.1%; for maternal adiponectin, 1.0% and 1.3%; and for cord adiponectin, 1.6% and 5.5%.

### 2.8 Determination of soluble leptin receptor concentrations

The plasma level of sLeptinR was measured by an ELISA commercial kit (Biorbyt, Cambridge, United Kingdom). The detection range of the ELISA assay was 15.6–1,000 pg/mL, and the sensitivity of the test was 9.4 pg/mL. All samples were diluted 250 times and assayed in duplicate. The intra- and interassay coefficients of variation were 1.3% and 4.5% for maternal plasma and 3.4% and 5.6% for cord blood plasma, respectively.

### 2.9 Leptin-to-adiponectin ratio and free leptin index

The leptin-to-adiponectin ratio, a composite biomarker reflecting adipose tissue dysfunction and metabolic risk, was calculated as follows:

LAR, plasma Leptin [ng/mL] /plasma Adiponectin [µg/mL].

The FLI, used to estimate the amount of biologically active (unbound) leptin in circulation relative to sLeptinR concentration, was calculated as follows:

FLI, plasma Leptin [ng/mL] / plasma Soluble Leptin Receptor [ng/mL].

### 2.10 Statistical analysis

Statistical analyses were performed using TIBCO STATISTICA, version 13.3 (StatSoft, Inc., Tulsa, OK, USA). Categorical variables, including maternal age (<35 vs. ≥35 years), BMI (normal weight, overweight, obesity), GA (near preterm birth, term birth), mode of delivery (vaginal, cesarean section), and newborn sex, were summarized as frequencies and percentages (*n*/*N*, %). Continuous variables were expressed as the median, mean ± standard deviation (SD), and interquartile range (25th–75th percentiles). Group comparisons for categorical data were performed using the chi-square test. The distribution of continuous variables was assessed with the Shapiro–Wilk test. Since most distributions deviated from normality, non-parametric tests were applied. For the calculation of statistical significance, the Kruskal–Wallis and Mann–Whitney *U* tests were used.

The strength and direction of monotonic associations among adipokines, as well as between adipokines and maternal or neonatal anthropometric parameters in the GDM and non-GDM (control) groups, were assessed using Spearman’s rank correlation. To control the False Discovery Rate (FDR) across perform tests and minimize false positives, we applied the Benjamini-Hochberg (BH) procedure.

FDR-corrected q-values are reported alongside uncorrected p-values. Results were present in Tables and in the form of heat maps, where dark red represented the strongest positive correlations and dark green the strongest negative correlations. A two-tailed q(p)-value <0.05 was considered as statistically significant.

## 3 Results

### 3.1 Characteristics of the study population

Fifty-three paired maternal plasma and umbilical cord blood plasma samples were collected from women with normoglycemia (*n* = 23) and those diagnosed with gestational diabetes mellitus (GDM-diet, *n* = 16; GDM-insulin, *n* = 14). Participant characteristics are summarized in **Table 1**. All plasma samples were collected from white European women. The study groups did not differ significantly in maternal anthropometric parameters (age, pre-pregnancy BMI), the prevalence of comorbidities (hypertension, hypothyroidism), obstetric factors (gestational age at delivery, parity, mode of delivery), or neonatal features (birth weight, gender, crown–heel length, head and chest circumference) that could potentially influence biochemical variable concentrations in the paired plasma samples.

**Table 1:** Characteristics of the study population.

Analyzed parameters	GDM *N* = 30 (% (*n*/*N*))	GDM-insulin *N* = 16 (% (*n*/*N*))	GDM-insulin *N* = 14 (% (*n*/*N*))	Non-GDM group *N* = 23 (% (*n*/*N*))	Chi-Square Test (GDM vs non-GDM) χ2	*p*-Value
Race/ethnicity • White European women, as self-reported	100% (30/30)	100% (16/16)	100% (14/14)	100% (23/23)	NA	NA
Women’s age (mean ± SD)	32.95 ± 4.20	33.53 ± 4.78	32.36 ± 3.61	31.96 ± 3.87	1.83	0.40
• 20–34	60.00% (18/30)	50.00% (8/16)	71.43% (10/14)	78.26% (18/23)		
• ≥35	36.67% (11/30)	43.75% (7/16)	28.57% (4/14)	26.09% (6/23)		
• no information	3.33% (1/30)	6.25% (1/16)	0.00% (0/14)	0.00% (0/23)		
Women’s BMI, kg/m^2^ (mean ± SD)	26.93 ± 4.65	25.61 ± 4.90	28.71 ± 4.00	23.88 ± 4.37	4.63	0.10
• Normal weight (18.5–24.9)	20.00% (6/30)	25.00% (4/16)	14.29% (2/14)	47.83% (11/23)		
• Overweight and obesity (OWO) (≥25–29.9)	26.67% (8/30)	25.00% (4/16)	28.57% (4/14)	17.39% (4/23)		
• No information	53.33% (16/30)	50.00%(8/16)	57.14% (8/14)	34.78% (8/23)		
Parity						
• 1	56.66% (17/30)	62.50% (10/16)	50.00% (7/14)	34.78% (8/23)	4.88	0.09
• 2	26.67% (8/30)	31.25% (5/16)	21.43% (3/14)	56.52% (13/23)		
• ≥3	16.67% (5/30)	6.25% (1/16)	28.57% (4/14)	8.70% (2/23)		
Gestational age, weeks (mean ± SD)	38.53 ± 1.20	38.69 ± 1.40	38.36 ± 1.00	39.00 ± 1.78		
• Near term: 35–37 weeks	16.67% (5/30)	18.76% (3/16)	14.29% (2/14)	17.39% (4/23)	0.0002	1.0
• Term: 38–42 weeks	80.00% (24/30)	81.25% (13/16)	85.71% (12/14)	82.61% (19/23)		
Delivery mode						
• Vaginal birth	13.33% (4/30)	6.25% (1/16)	21.43% (3/14)	13.04% (3/23)	0.001	0.99
• Cesarean section	86.67% (26/30)	93.75% (15/16)	78.57% (11/14)	86.97% (20/23)		
Mother’s diseases						
• Hypertension	16.67% (5/30)	12.50% (2/16)	21.43% (3/14)	17.39% (4/23)	0.21	0.65
• Hypothyroidism	16.67% (5/30)	18.75% (3/16)	14.29% (2/14)	26.09% (6/23)		
Birth weight, g (mean ± SD)	3,302 ± 532	3,276 ± 486	3,326 ± 577	3,398 ± 546	2.03	0.36
• Small for gestational age (SGA)	16.67% (5/30)	18.75% (3/16)	14.29% (2/14)	8.70% (2/23)		
• Appropriate for gestational age (AGA)	76.67% (23/30)	81.25% (13/16)	71.43% (10/14)	69.56% (16/23)		
• Large for gestational age (SGA)	6.67% (2/30)	0.00% (0/16)	14.29% (2/14)	17.39% (4/23)		
Newborns’ gender						
• Male	30.00% (9/30)	31.25% (5/16)	28.57% (4/14)	43.48% (10/23)	2.41	0.30
• Female	53.33% (16/30)	62.50% (10/16)	42.86% (6/14)	52.17% (12/23)		
• No information	16.67% (5/30)	6.25% (1/16)	28.57% (4/14)	4.35% (1/23)		
Crown–heel length, cm (mean ± SD)	53 ± 4	53 ± 3	53 ± 4	53 ± 3	NA	NA
Head circumference, cm (mean ± SD)	37 ± 2	35 ± 1	35 ± 2	35 ± 1		
Chest circumference, cm (mean ± SD)	33 ± 2	33 ± 2	34 ± 2	34 ± 2		

The table shows values, which are given as means ± SDs (ranges) and the percentage values, representing the number of milk donors in the given subgroup (*n*) in relation to all milk donors (N), for whom the specific information was available. NA, not analyzed.

The mean maternal age in the GDM group was 32.95 ± 4.20 years, which was slightly higher than in the non-GDM group (31.96 ± 3.87 years). Pre-pregnancy BMI values were higher in women with GDM. Specifically, BMI was 25.61 ± 4.90 kg/m² in GDM-diet and 28.71 ± 4.00 kg/m² in GDM-insulin, compared to 23.88 ± 4.37 kg/m² in the non-GDM cohort; however, these differences were not statistically significant. Data on maternal pre-pregnancy BMI values were missing for 53.33% of women in the GDM group and 34.78% in the non-GDM group (**Table 1**). Comorbidities were observed at similar rates in both groups. Hypertension was present in 16.67% of women with GDM compared to 17.39% in the non-GDM group. Hypothyroidism was diagnosed in 16.67% of the GDM group and in 26.09% of the non-GDM group.

Most women in all study groups delivered between 38 and 42 weeks of gestation (GDM-diet: 81.25%, GDM-insulin: 85.71%, non-GDM: 82.61%). Cesarean section was the predominant mode of delivery, accounting for 86.67% of births in both GDM and non-GDM groups (**Table 1**). Regarding parity, 56.66% of women in the GDM group were primiparous, whereas in the non-GDM group, most participants had two prior deliveries.

Neonatal birth weight did not differ significantly between groups, averaging 3276 ± 486 g (GDM-diet), 3326 ± 577 g (GDM-insulin), and 3398 ± 546 g (non-GDM) (**Table 1**). The proportion of female newborns was 62.50% in GDM-diet, 42.86% in GDM-insulin, and 52.17% in the non-GDM group. Data on neonatal sex were missing for 16.67% of infants in the GDM group and 4.35% in the non-GDM group.

With respect to growth status, birth weight AGA was observed in 81.25% of infants in the GDM-diet group, 71.43% in GDM-insulin, and 69.56% in the non-GDM group. SGA newborns accounted for 16.67% in the GDM group and 8.70% in the non-GDM group, whereas LGA infants were observed in 6.67% and 17.39% of these groups, respectively (**Table 1**).

The mean crown–heel length was 53 ± 4 cm in the GDM group and 53 ± 3 cm in the non-GDM group. Similarly, the mean head circumference measured 35 ± 2 cm in newborns of mothers with GDM and 35 ± 2 cm in the non-GDM group. The mean chest circumference was 33 ± 2 cm in the GDM group and 34 ± 2 cm in the non-GDM group (**Table 1**).

### 3.2 Maternal plasma profiles of leptin, adiponectin, sLeptinR, LAR, and FLI in the GDM and non-GDM groups

The median maternal leptin concentration was significantly higher in the GDM-insulin group (10.05 ng/mL) compared to the non-GDM group (4.44 ng/mL) (*p* = 0.033) (**Table 2**). In the GDM-diet group, leptin concentration was moderately elevated (5.59 ng/mL) relative to the non-GDM group; however, this difference did not reach statistical significance. No statistically significant difference was found in adiponectin concentration among the analyzed groups in relation to the severity of maternal hyperglycemia (GDM-diet: 2.09 µg/mL, GDM-insulin: 1.61 µg/mL, and non-GDM: 1.79 µg/mL) (**Table 2**).

**Table 2:** Adiponectin–leptin axis and indicators in GDM maternal plasma.

Analyzed parameters	GDM *N* = 30 (% (*n*/*N*))	GDM-diet *N* = 16 (% (*n*/*N*))	GDM-insulin *N* = 14 (% (*n*/*N*))	Non-GDM group *N* = 23 (% (*n*/*N*))	*p*-value^a^		*p*-value^b^	
						diet vs. insulin	diet vs. non-GDM	insulin vs. non-GDM
Leptin, ng/mL								
Mean ± SD	7.31 ± 5.26	5.86 ± 4.96	8.97 ± 5.27	5.15 ± 3.24	0.17	0.08	0.81	0.033
Median	6.04	5.59	10.05	4.44				
25–75 percentiles	2.88–11.49	2.12–6.62	3.68–12.47	3.23–6.73				
Adiponectin, μg/mL								
Mean ± SD	1.97 ± 0.72	2.16 ± 0.58	1.76 ± 0.81	1.90 ± 0.76	0.26	0.12	0.23	0.90
Median	1.93	2.09	1.61	1.79				
25–75 percentiles	1.48–2.55	1.80–2.58	1.19–2.15	1.37–2.59				
Leptin-to-adiponectin ratio (LAR)								
Mean ± SD	4.91 ± 4.74	3.66 ± 4.31	6.34 ± 4.95	3.69 ± 4.16	0.37	0.043	0.79	0.057
Median	3.49	2.18	4.99	2.43				
25–75 percentiles	1.26–7.29	1.00–4.04	2.03–9.55	1.46–4.30				
sLeptinR, ng/mL								
Mean ± SD	59.47 ± 14.88	63.13 ± 16.67	55.30 ± 11.74	53.55 ± 12.33	0.17	0.22	0.06	0.75
Median	57.54	61.08	56.35	53.75				
25–75 percentiles	50.26–68.11	52.73–72.27	42.13–63.09	47.26–60.07				
Free leptin index (FLI)								
Mean ± SD	0.17 ± 0.14	0.13 ± 0.14	0.21 ± 0.12	0.20 ± 0.19	0.16	0.058	0.20	0.56
Median	0.13	0.08	0.20	0.12				
25–75 percentiles	0.05–0.25	0.04–0.16	0.10–0.30	0.05–0.35				

Values are given as mean ± SD, median, and 25th–75th percentiles. The Kruskal–Wallis and Mann–Whitney *U* tests were used for statistical calculations, and a *p*-value lower than 0.05 was regarded as significant, marked in red. LAR, leptin-to-adiponectin ratio; sLeptinR, soluble leptin receptor.

^a^Kruskal–Wallis test.

^b^Mann–Whitney *U* test.

The LAR was higher in the GDM group (median 3.49) compared to the non-GDM group (2.43), but this difference was not statistically significant. A statistically significant difference in LAR was found between the GDM-diet (2.18) and GDM-insulin (4.99) subgroups (*p* = 0.043). The LAR in the GDM-insulin group nearly reached statistical significance when compared to the non-GDM group (*p* = 0.057; **Table 2**).

The concentration of sLeptinR in maternal plasma was not significantly higher in the GDM-diet group (61.08 ng/mL) and the GDM-insulin group (56.35 ng/mL) compared to the non-GDM group (53.75 ng/mL) (**Table 2**). The free leptin index was elevated in the GDM-insulin group (0.20) compared to GDM-diet (0.08; *p* = 0.058, approaching significance), though no significant differences were observed between either GDM subgroup and the non-GDM group (**Table 2**).

### 3.3 Cord blood plasma profiles of leptin, adiponectin, sLeptinR, LAR, and FLI in the GDM and non-GDM groups

Leptin concentrations in cord blood plasma did not differ significantly between GDM-diet (4.37 ng/mL) and GDM-insulin (4.07 ng/mL) subgroups. Median leptin levels were higher in both GDM subgroups than in the non-GDM group (3.56 ng/mL), but these differences were not statistically significant (**Table 3**).

**Table 3:** Adiponectin–leptin axis and indicators in neonatal cord plasma by GDM status.

Analyzed parameters	GDM *N* = 30 (% (*n*/*N*))	GDM-diet *N* = 16 (% (*n*/*N*))	GDM-insulin *N* = 14 (% (*n*/*N*))	Non-GDM group *N* = 23 (% (*n*/*N*))	*p*-value^a^	*p*-value^b^		
						diet vs. insulin	diet vs. non-GDM	insulin vs. non-GDM
Leptin, ng/mL								
Mean ± SD	7.73 ± 8.82	6.95 ± 7.15	8.62 ± 10.64	5.91 ± 4.69	0.89	0.79	0.88	0.36
Median	4.07	4.37	4.07	3.56				
25–75 percentiles	2.02–9.81	2.01–9.27	2.62–10.22	2.12–9.60				
Adiponectin, μg/mL								
Mean ± SD	7.78 ± 3.69	9.29 ± 3.83	6.06 ± 2.74	6.68 ± 2.63	0.02	0.01	0.02	0.43
Median	7.27	9.05	5.70	6.01				
25–75 percentiles	5.51–9.68	7.08–11.87	4.10–7.31	5.40–8.56				
Leptin-to-adiponectin ratio (LAR)								
Mean ± SD	1.13 ± 1.22	0.81 ± 0.84	1.49 ± 1.50	1.21 ± 1.87	0.35	0.19	0.39	0.21
Median	0.80	0.64	1.13	0.56				
25–75 percentiles	0.24–1.34	0.24–0.97	0.36–2.23	0.34–1.49				
sLeptinR, ng/mL								
Mean ± SD	35.40 ± 5.90	36.81 ± 5.47	33.78 ± 6.15	38.56 ± 10.87	0.32	0.31	0.53	0.27
Median	34.79	35.68	34.35	41.27				
25–75 percentiles	32.42–39.31	32.36–39.89	32.42–36.91	30.89–43.82				
Free leptin index (FLI)								
Mean ± SD	0.22 ± 0.26	0.20 ± 0.23	0.25 ± 0.31	0.16 ± 0.12	0.83	0.70	0.81	0.33
Median	0.12	0.14	0.12	0.08				
25–75 percentiles	0.05–0.27	0.05–0.26	0.09–0.31	0.07–0.27				

Values are given as mean ± SD, median, and 25th–75th percentiles. The Kruskal–Wallis and Mann–Whitney *U* tests were used for statistical calculations, and a *p*-value lower than 0.05 was regarded as significant, marked in red. LAR, leptin-to-adiponectin ratio; sLeptinR, soluble leptin receptor.

^a^Kruskal–Wallis test.

^b^Mann–Whitney *U* test.

In contrast, the concentration of adiponectin in cord blood plasma was significantly higher in the overall GDM group (7.27 µg/mL) compared to the non-GDM group (6.01 µg/mL; *p* = 0.02). Significant differences in adiponectin levels were found between GDM-diet and GDM-insulin (9.05 vs. 5.70 µg/mL; *p* = 0.01) and between the GDM-diet and non-GDM groups (9.05 vs. 6.01 µg/mL; *p* = 0.02). No significant difference was observed between GDM-insulin and non-GDM cohorts (5.70 µg/mL vs. 6.01 µg/mL; *p* = 0.43) (**Table 3**).

LAR values were higher in GDM-diet (0.64) and GDM-insulin (1.13) subgroups than in the non-GDM group (0.56), but these differences were not statistically significant (*p* = 0.19; **Table 3**).

The concnetration of sLeptinR in cord blood plasma was not significantly influenced by maternal glycemic status (GDM-diet: 35.68 ng/mL; GDM-insulin: 34.35 ng/mL; both non-significantly lower than non-GDM: 41.27 ng/mL). Similarly, the median FLI did not differ significantly according to the severity of hyperglycemia, being 0.14 for GDM-diet, 0.12 for GDM-insulin, and 0.08 for non-GDM (**Table 3**).

### 3.4 Correlations of adipokines with maternal and neonatal outcomes

**Figure 1:**
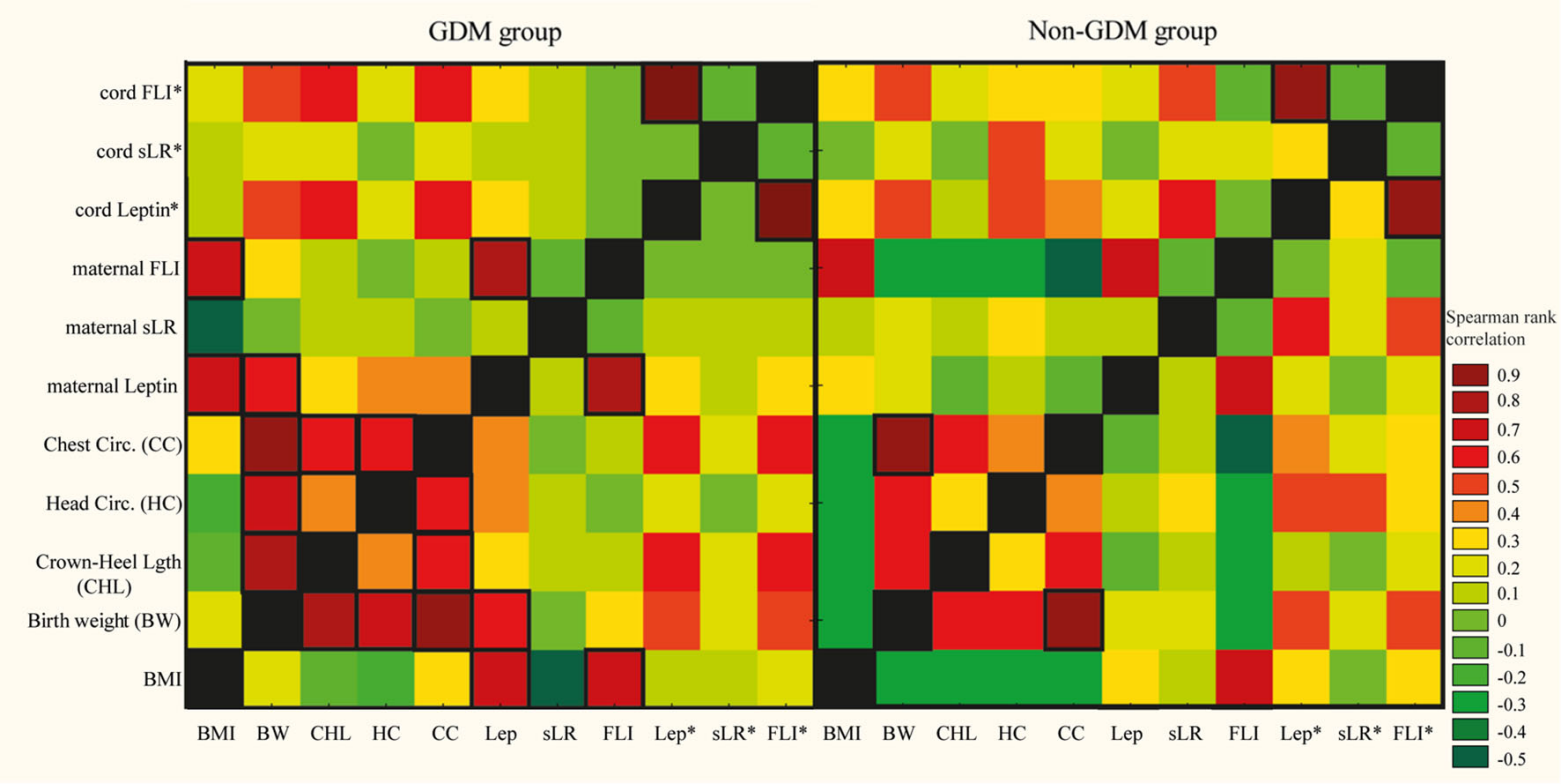
Correlations between the concentration of adipokine in maternal and cord plasma collected from GDM and non-GDM mothers and body mass index (BMI), birth weight (BW), crown–heel length (CHL), head circumference (HC), chest circumference (CC), leptin (Lep), soluble leptin receptor (sLR), free leptin index (FLI). The Spearman correlation coefficient is represented in the heat map following the color in the legend. To control for false associations, the Benjamini-Hochberg correction was applied. Bold frames represent correlations with statistical significance (*q* < 0.05). *cord blood plasma.

To assess the relationships between adipokine concentrations in maternal and cord blood plasma and maternal pre-pregnancy BMI, newborn anthropometric parameters—including birth weight, crown–heel length, and head and chest circumference, Spearman correlation coefficients were calculated. Due to the lack of significant differences for leptin, sLeptinR, and FLI levels between GDM-diet and GDM-insulin mothers, these two cohorts were merged together (**Figure 1**, **Supplementary Table S1**). Adiponectin concentration and LAR values in maternal and cord blood plasma were analyzed in relation to the severity of maternal hyperglycemia (**Table 4**).

**Table 4:** Adiponectin–LAR correlations across maternal and cord plasma by GDM status.

Analyzed fluid		Adiponectin			Leptin-to-Adiponectin Ratio (LAR)		
		GDM-diet	GDM-insulin	Non-GDM	GDM-diet	GDM-insulin	Non-GDM
Maternal plasma	BMI	-0.36	**-0.85, *p*=0.03** ***q*=0.39**	-0.03	0.67	**0.84, *p*=0.04** ***q*=0.39**	0.08
	Birth Weight	0.10	-0.33	-0.19	0.28	**0.81, *p*=0.0003** ***q*=0.01**	0.37
	Crown-heel length	0.28	0.40	-0.08	0.24	0.18	0.08
	Head circumference	0.04	-0.29	-0.32	0.15	0.56	0.08
	Chest circumference	-0.19	-0.19	-0.11	0.30	**0.72, *p*=0.02** ***q*=0.39**	0.11
	Leptin	0.03	-0.17	-0.39	NA	NA	NA
	sLeptinR	0.10	0.34	0.21	0.42	-0.40	0.05
	FLI	-0.07	-0.07	0.04	NA	NA	NA
Cord blood plasma	BMI	-0.31	0.18	0.09	0.01	0.23	-0.07
	Birth Weight	**0.51, *p*=0.047** ***q*=0.39**	-0.01	0.14	0.06	0.38	**0.48, *p*=0.02** ***q*=0.39**
	Crown-heel length	**0.55, *p*=0.034** ***q*=0.39**	-0.30	-0.01	-0.20	0.51	0.38
	Head circumference	**0.53, *p*=0.044** ***q*=0.39**	0.05	0.06	-0.15	0.13	0.35
	Chest circumference	0.49	0.10	0.14	0.06	0.27	0.41
	Leptin	0.14	0.39	0.11	NA	NA	NA
	sLeptinR	0.15	0.17	-0.36	-0.33	0.03	**0.63, *p*=0.002** ***q*=0.06**
	FLI	0.11	0.38	0.27	NA	NA	NA

Spearman's rank correlation was used for pairwise comparisons of adiponectin and LAR in maternal and cord blood plasma across GDM subgroups (GDM-diet and GDM-insulin) and the control group (non-GDM), with women's and neonatal anthropometric parameters, as well as leptin, sLeptinR, and FLI. Data represent targeted pairwise Spearman correlations within each group. Spearman coefficients are presented with uncorrected p-values and FDR-corrected q-values (Benjamini-Hochberg procedure). Significance thresholds: p < 0.05(uncorrected, bold) and q < 0.05 (FDR-corrected, bold and red). BMI, Body Mass Index; FLI, Free Leptin Index; sLeptinR, soluble Leptin Receptor; Non-GDM, normoglycemic group; GDM-diet, diet-treated GDM; GDM-insulin, insulin-treated GDM; NA, not analazyed.

**Figure 2:**
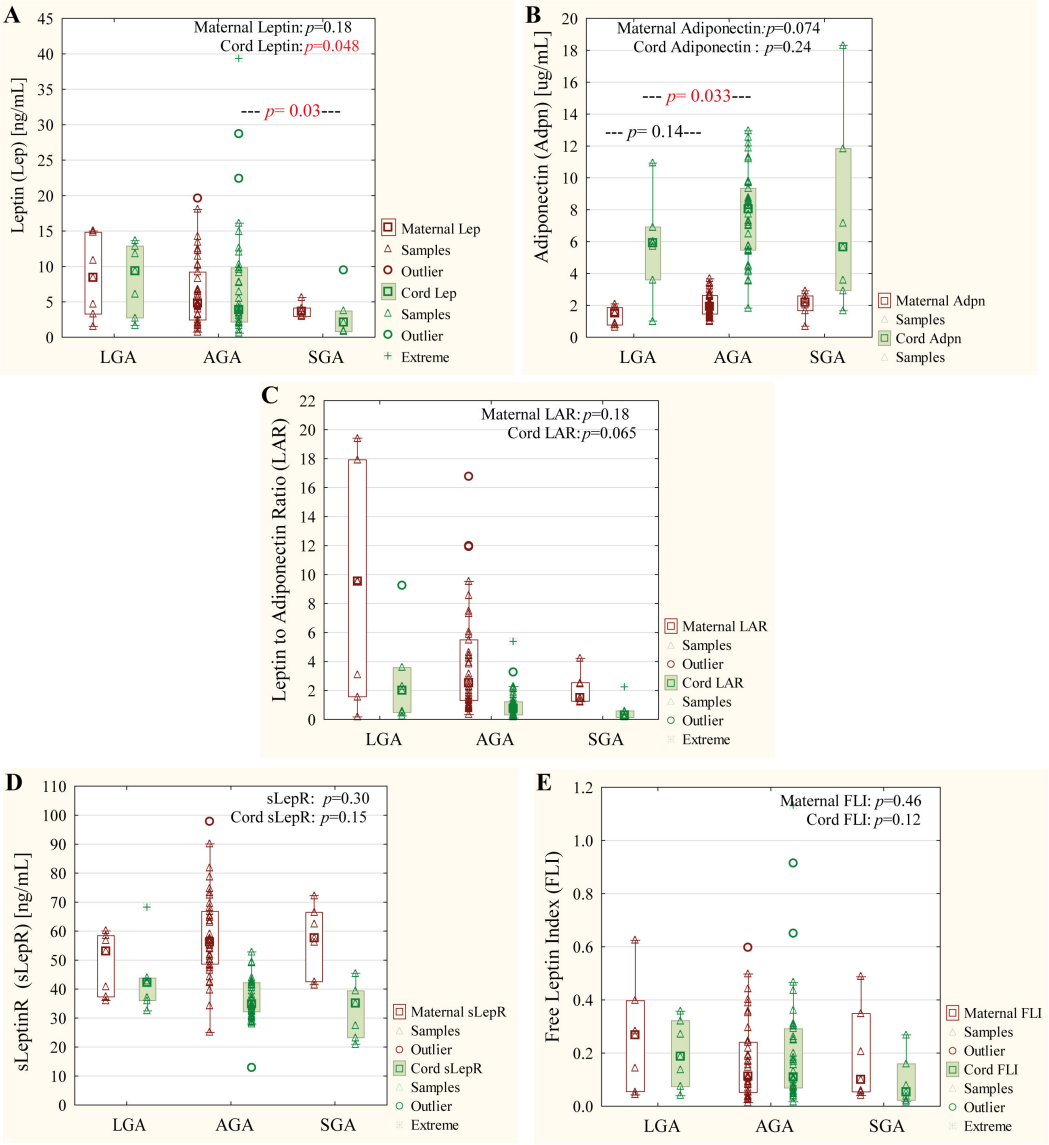
Panels **(A–E)** present maternal plasma leptin **(A)**, adiponectin **(B)**, leptin–adiponectin ratio (LAR, C), soluble leptin receptor (sLeptin R, D) and free leptin index (FLI, E) according to neonatal birth weight. Data are given as mean and median values and 25th and 75th quartiles. A p-value lower than 0.05 was regarded as significant. AGA, appropriate for gestational age; LGA, large for gestational age; SGA, small for gestational age. Kruskal–Wallis test was used for statistical calculations, and a p-value lower than 0.05 was regarded as significant and was marked red color.

In the GDM cohort, moderate correlation was observed between maternal plasma leptin (*r* = 0.61, *q*=0.01) concentration and maternal BMI, whereas such association was absent in the non-GDM group. Within the GDM cohort, positive correlations were found between maternal leptin and newborn birth weight (*r*=0.51, *q*<0.04). In the non-GDM group, maternal plasma leptin did not show correlation with newborn birth weight (**Figure 2**, **Supplementary Table 1**).

Strong positive correlation was observed between maternal plasma LAR (but not adiponectin) and neonatal weight in the GDM-insulin group (*r* = 0.81, *q* = 0.01). Strong correlations were found between maternal plasma adiponectin as well as LAR levels and women’s BMI in the GDM-insulin group (*r* = -0.85 (*p* = 0.03) and *r* = 0.84 (*p* = 0.04), respectively), however these associations were not statistically significant after applied Benjamini-Hochberg correction (*q* = 0.39 and *q* = 0.39, respectively). No associations were observed in the GDM-diet or non-GDM groups (**Table 4**).

Cord plasma adiponectin showed a moderate positive correlation with neonatal anthropometric measurements, including birth weight (*r* = 0.51, *p* = 0.047), crown–heel length (*r* = 0.55, *p* = 0.034), and head circumference (*r* = 0.53, *p* = 0.044) in GDM-diet group, however, these were not statistically significant after Benjamini-Hochberg correction (*q* = 0.39 for all associations). In the non-GDM group, cord plasma LAR showed a positive correlation with birth weight (*r* = 0.48, *p* = 0.02) that was lost after correction (*q* = 0.39), whereas sLeptinR levels were positively associated with LAR (*r* = 0.63, *p* = 0.002), with a *q* value that approached statistical significance (*q* = 0.06) (**Table 4**).

### 3.5 Maternal and cord blood plasma profiles of leptin, adiponectin, sLeptinR, LAR, and FLI in relation to the LGA, AGA, and SGA groups

Cord blood plasma leptin levels varied significantly between the birth-size categories (*p* = 0.048), with the highest concentrations observed in the LGA group (median: 9.38 ng/mL; mean: 8.32 ± 4.87 ng/mL), followed by AGA (median: 3.87 ng/mL; mean: 7.43 ± 8.02 ng/mL) and SGA (median: 2.12 ng/mL; mean: 2.83 ± 3.09 ng/mL) (*p* =0.048; **Figure 2A**). No significant differences in maternal plasma leptin levels were observed across neonatal birth categories (*p* = 0.18; **Figure 2A**). For maternal plasma adiponectin, but not for cord blood plasma, a significant difference was found between the LGA (median: 1.52 µg/mL, mean: 1.33 ± 0.56 µg/mL) and AGA (median: 1.93 µg/mL, mean: 2.03 ± 0.72 µg/mL) groups (*p* = 0.033). No significant differences were detected in the concentrations of sLeptinR, LAR, or FLI in either maternal or cord blood plasma across the birth−size categories (**Figures 2C–E**).

## 4 Discussion

Maternal health before and during pregnancy is crucial for optimal fetal development and the wellbeing of both the mother and child. Maternal preconception factors, including weight and preexisting health status, influence both short- and long-term pregnancy outcomes. Effective management of these variables before conception is crucial to improving maternal and neonatal health. This study addresses critical gaps in knowledge regarding the impact of maternal factors on fetal metabolic programming, with a special focus on adipokine homeostasis during the perinatal period. We identified significant differences in the concentrations of leptin, adiponectin, and the leptin-to-adiponectin ratio in the maternal–fetal axis, which was associated with the severity of maternal hyperglycemia, classified as GDM-diet and GDM-insulin. Moreover, we identified an association between cord blood leptin levels and birth-size classification. These findings have the potential to improve early risk assessment for metabolic disorders and to guide the development of targeted preventive and nutritional interventions for women at high risk. Ultimately, our results contribute to advancing strategies for early detection and prevention of metabolic health issues in offspring, supporting better maternal and child health outcomes.

### 4.1 GDM and adipokine-based metabolic factors

Exposure to GDM affects maternal and neonatal adipokine profiles, particularly leptin and adiponectin concentrations. Overall, on the second day after delivery, maternal plasma leptin level was higher in the GDM group (6.04 ng/mL) than in the non-GDM group (4.44 ng/mL); however, this difference did not reach statistical significance. This observation aligns with the findings of Chen et al. (63), who reported that leptin concentrations differed significantly between GDM and non-GDM, before, but not after, delivery. Interestingly, in our study, maternal plasma leptin concentrations were significantly elevated in the insulin-treated GDM subgroup (10.05 ng/mL) compared to the non-GDM group (4.44 ng/mL), emphasizing the influence of impaired glucose homeostasis on leptin regulation. Cord blood leptin level was higher in infants born to mothers with GDM (4.07 ng/mL) than in those from the non-GDM group (3.56 ng/mL), but this difference was not statistically significant, which is in line with the data presented by Gruber et al. (77). In contrast to maternal plasma, we did not find a significantly higher concentration of cord leptin for GDM-insulin in comparison to the non-GDM group. The lack of statistically significant differences in cord plasma leptin concentration in our study may be attributable to high standard deviation observed, which likely reflect variability in maternal anthropometric parameters such as body mass index (**Supplementary Figure 1**) and gestational weight gain (the variable not recorded in the study) (78).

In our study, we did not observe significantly lower maternal plasma adiponectin concentrations in GDM subgroups compared to the control group, as previously reported (77, 79, 80). However, it should be noted that most adiponectin analyses were conducted during the first or second trimester of pregnancy, whereas in this study, adiponectin was measured on postpartum day 2. Pala et al. (81) showed that maternal adiponectin increases after delivery and found no significant differences in adiponectin levels between GDM and control groups in the postpartum period, consistent with our observations. On the other hand, Thyfault et al. (82) demonstrated that adiponectin concentrations depend on the severity of insulin resistance and carbohydrate metabolism disorders. This may at least partially explain the strong negative correlation we observed between maternal plasma adiponectin concentration and BMI in the GDM-insulin group, however, this association was statistically significant only before Benjamini-Hochberg correction.

We identified a significant difference in cord blood adiponectin levels between diet-treated GDM pregnancies and the non-GDM group (9.05 µg/mL and 6.01 µg/mL; *p* = 0.02; respectively), whereas no significant difference was found for insulin-treated GDM pregnancies compared to non-GDM. Presented by us values for cord blood adiponectin concentration align with those presented by Aramesh et al. (83). The authors reported significantly higher mean cord blood adiponectin concentrations in the GDM group (11.05 ± 4.1 µg/mL) compared to the normoglycemic group (5.34 ± 2.63 µg/mL); however, it should be highlighted that the severity of maternal hyperglycemia was not recorded in this study (83). There was a higher concentration of cord blood adiponectin in infants born to mothers with GDM-diet compared to non-GDM, suggesting that even mild maternal hyperglycemia can alter fetal adiponectin dynamics. It was reported that adiponectin enhances insulin sensitivity and exerts anti-inflammatory effects (84). Observed by us alterations in cord adiponectin pattern could disrupt nutrient partitioning and lipid metabolism in utero, potentially increasing metabolic vulnerability and the risk of obesity and insulin resistance later in life (11, 85).

For LAR, a significant difference was observed between the values for maternal plasma of the GDM-diet and GDM-insulin groups (2.18 and 4.99, respectively; *p* = 0.043), but not for cord blood plasma. Our findings suggest that maternal metabolic alterations are more prominently reflected in maternal plasma than in cord blood plasma. In addition, LAR was significantly higher in the insulin-treated GDM group, suggesting more pronounced metabolic dysregulation and a potentially increased risk of developing cardiometabolic complications later in life.

The concentration of sLeptinR in maternal plasma ranged from 56.35 ng/mL (non-GDM) to 61.08 ng/mL (GDM-diet) and was higher than in cord blood plasma (GDM-insulin: 34.79 ng/mL and non-GDM: 41.27 ng/mL), and this trend is in line with the data shown by other authors (63, 86). Our postpartum plasma sLeptinR values (day 2) align with the data presented by Chen et al. (63) for day 3 after postpartum but exceed the values presented by Marino-Ortega et al. (62) (20.2–25.9 ng/mL, samples collected immediately after delivery), primarily due to differences in sampling time points. Interestingly, in our cohort, maternal plasma sLeptinR concentrations were higher in diet-treated GDM (61.08 ng/mL) than in non-GDM controls (53.75 ng/mL; *p* = 0.06), approaching significance, while no such elevation occurred in insulin-treated GDM (56.35 ng/mL). As was reported (87, 88), this borderline trend warrants consideration. It suggests compensatory sLeptinR upregulation in milder, diet-controlled GDM to counter hyperleptinemia and preserve leptin sensitivity, a mechanism potentially disrupted by severe metabolic dysregulation in insulin-requiring cases. Although maternal hyperleptinemia is typically associated with increased GDM risk (89), elevated concentrations of sLeptinR may reduce leptin bioavailability by sequestering circulating leptin, thus attenuating its biological activity and potentially contributing to a more favorable metabolic profile (90). In cord blood samples, no statistically significant differences were observed for sLeptinR between the GDM and non-GDM groups.

In our study, a higher maternal plasma FLI was observed for the insulin-treated group (0.20) compared to the diet-treated group (0.08), with the *p*-value approaching significance (*p* = 0.058). To the best of our knowledge, this is the first study that has examined FLI status in the maternal–fetal axis in relation to the severity of maternal hyperglycemia. Previously, Mosavat et al. (86) reported data on leptin and sLeptinR concentration in GDM and non-GDM samples collected; however, they did not analyze FLI in their study. Nevertheless, their findings suggest FLI on a comparable level, which was also observed in our study for the GDM and non-GDM cohorts (0.13 and 0.12, respectively). This higher value of FLI for mothers with insulin-treated hyperglycemia during pregnancy may indicate reduced leptin sensitivity and increased metabolic stress. Owecki et al. (91) reported that elevated FLI values have been strongly correlated with subsequent metabolic abnormalities and may serve as a superior predictor of type 2 diabetes risk compared to conventional biomarkers. On the other hand, Gajewska et al. (92) analyzed FLI in Polish healthy children and adolescents and found that alterations in adipokine profile (including FLI) can result in a predisposition to the development of obesity and obesity-related complications. Furthermore, Kimber-Trojan et al. (93) proposed that the SFRP5/leptin ratio together with FLI, might be early signals of metabolic dysregulation in women exposed on GDM. In our study, no significant differences in FLI were observed in cord blood plasma. Hytinantti et al. (94) reported significantly higher concentrations of free leptin in cord plasma from infants born to mothers with GDM compared to those of healthy mothers; however, they employed a different methodological approach, analyzing free and bound leptin concentrations using high−performance liquid chromatography rather than the leptin-to-sLeptinR index (FLI). The maternal–fetal FLI data presented here underscore the placenta’s selective regulation of leptin transfer and metabolism, safeguarding fetal energy homeostasis amid maternal metabolic perturbations.

### 4.2 Correlation of maternal and cord plasma adipokines with maternal metabolic state and neonatal anthropometric outcomes

In this study, the observed moderate correlations between maternal plasma leptin and sLeptinR levels and maternal BMI in the GDM cohort align with previous data (62, 95). Several studies report elevated maternal leptin concentrations in GDM, which correlate positively with maternal BMI and insulin resistance, reflecting leptin’s role as an adiposity marker and metabolic regulator during pregnancy (96–98). In contrast, in this study, these correlations were not observed in the non-GDM group, which underscores metabolic distinctions between the analyzed populations (**Figure 1**).

Previously report show that FLI was significantly increased in mild pre-eclamptic pregnancies and it allow to suggest that this rise can alter leptin bioavailability and bioactivity and thus might be associated with sympathetic hyperactivity and the hypertensive disorders during pregnancy (68). Moreover, we present associations between the analyzed variables and adiponectin/LAR levels in maternal and cord plasma across GDM management subgroups (GDM-diet and GDM-insulin). Due to the large number of correlations performed, most associations shown in **Table 4** lost statistical significance after Benjamini-Hochberg correction, which minimizes false-positive results. However, it is noteworthy that the associations between adiponectin and neonatal anthropometric measurements in the GDM-diet group (before p-value correction) remain consistent with prior findings linking adiponectin to higher birth weight in GDM, but not normoglycemic, pregnancies.

### 4.3 Adipokine- based metabolic factors and newborns weight

This study also presents data on leptin, adiponectin, sLeptinR, FLI, and LAR across the maternal–fetal interface, analyzed in relation to neonatal anthropometric outcomes. Our results revealed a strong association between cord blood leptin concentration and birth−size classification: the lowest concentrations in SGA infants (2.12 ng/mL), intermediate levels in AGA infants (3.87 ng/mL), and the highest in LGA newborns (9.38 ng/mL). This gradient supports leptin’s established role as a marker of fetal adiposity and growth trajectory, consistent with earlier reports (99–103). Higher cord leptin in LGA infants likely reflects increased adipose tissue development and enhanced placental leptin transfer under nutrient−rich conditions. On the other hand, low leptin concentration in cord blood plasma SGA newborns points to reduced fat stores secondary to placental insufficiency, intrauterine growth restriction, or undernutrition, impairing energy homeostasis and promoting postnatal catch-up growth. Leptin acts as a fetal growth factor; its deficiency is linked to lower IGF-1 and heightened long-term metabolic risks like obesity (102, 104, 105). Collectively, these findings reinforce leptin’s function as both a growth indicator and a mediator of developmental programming that influences postnatal metabolic adaptation.

Consistent with Lindberger et al. (106), in this study, maternal plasma adiponectin was inversely associated with LGA births, showing significantly lower concentrations than in AGA neonates (1.52 vs. 2.02 µg/mL; *p* = 0.033). Although maternal BMI may partly drive this relationship, previous evidence also suggests sex−specific effects, particularly in female infants (106); such differences, however, were not confirmed in the analyzed cohort. Makker et al. (102) reported an inverse association between cord adiponectin and LGA status only in preterm infants.

The interplay between maternal glycemic status, fetal adipokine milieu, and growth outcomes supports the concept of hyperglycemia−driven metabolic programming. Elevated intrauterine leptin exposure coupled with diminished adiponectin signaling may promote leptin resistance and impaired hypothalamic appetite regulation (107–109). Over time, this imbalance can increase susceptibility to obesity and insulin resistance (110). Notably, Lausten−Thomsen et al. (111) and Viswanathan et al. (110) reported that even in non−diabetic pregnancies, maternal obesity and nutrient oversupply induced similar adipokine imbalances, suggesting that leptin−related dysregulation is not limited to GDM.

An unfavorable adipokine profile—marked by elevated leptin and reduced adiponectin—is closely linked to central adiposity and early metabolic syndrome in children (112, 113). LAR effectively integrates these opposing hormonal cues into a single index of insulin resistance risk. Although LAR differences across birth-size groups lacked statistical significance, the near significant trend (*p* = 0.065) merits validation in larger cohorts.

Meanwhile, neither sLeptinR nor FLI differed significantly between groups, though both trended downward with decreasing birth weight. Such gradients may reflect reduced leptin transport or receptor responsiveness in smaller neonates, further implicating impaired leptin sensitivity in fetal growth restriction.

### 4.4 Maternal obesity and adipokine based metabolic factors

Despite incomplete maternal BMI data for full mother–newborn pairs in our cohort, we conducted additional analyses accounting for abnormal fat accumulation. Maternal overweight and obesity markedly alter adipokine profiles, especially leptin, with implications for maternal metabolism and infant development (114, 115). Maternal plasma leptin was significantly higher in overweight/obese women (6.21 ng/mL) than normal-weight controls (3.77 ng/mL; *p* = 0.04), confirming hyperleptinemia as a hallmark of adiposity-driven dysregulation (116–118). This reflects expanded adipose tissue secretion and placental production, which contribute to endothelial dysfunction, systemic inflammation, and heightened cardiometabolic risk (119). Cord leptin trended higher in offspring of overweight/obese mothers (8.44 vs. 3.22 ng/mL; *p* = 0.18), suggesting early fetal adaptation to nutrient excess and potential leptin resistance programming, though statistical significance was not reached. Adiponectin levels stayed stable across BMI groups, consistent with pregnancy’s protective insulin sensitivity mechanisms (120, 121). LAR and FLI rose significantly in maternal plasma (*p* = 0.009 and *p* = 0.007) but not in cord blood, underscoring placental gating of fetal exposure (102, 107, 108, 122, 123). Unchanged concentration of soluble leptin receptor reinforces leptin’s direct role in metabolic shifts. It suggests that the placenta buffers fetal leptin amid maternal stress. We hypothesize that lactation diets like vegan or Mediterranean—rich in unsaturated fats and polyphenols—may counteract pregnancy-related hyperleptinemia, restoring adipokine balance and supporting long-term metabolic health (114, 124).

### 4.5 Strengths and limitations

A major strength of this study lies in its rigorous methodological design and the application of well-validated GDM diagnostic criteria. This consistency ensures diagnostic comparability with international studies and enhances the reproducibility of findings. Moreover, the carefully balanced recruitment protocol accounted for gestational age confounders, which could otherwise obscure adipokine associations. Such methodological precision allowed for a clearer assessment of how disturbances in maternal glycemic status contribute to leptin-adiponectin axis across the maternal–fetal interface. By simultaneously measuring maternal and cord plasma metabolic paramethers, the study also provides novel insight into mother–child metabolic cross-talk at the perinatal period.

Nonetheless, several limitations warrant consideration when interpreting these results. The data were analyzed from a single center only, and the lack of a validation cohort from another hospital in a different geographical region limits the generalizability of our findings. Moreover, it should be noted that cord blood and maternal blood were not collected on the same day. Other limitations include the unavailability of complete preconception BMI values (due to the limited samples with available BMI data, mothers suffering from higher fat accumulation, such as overweight and obesity, were merged into the OWO group) and the lack of maternal gestational weight gain data, which precluded assessment of their contribution to the adipokine profile among the analyzed variables. Excessive gestational weight gain has been shown to amplify oxidative stress and adipose-derived cytokine release, potentially intensifying metabolic imbalance during pregnancy. The absence of HbA1c measurements also limited the exclusion of women with undiagnosed pregestational diabetes, possibly affecting baseline glycemic context and adipokine variability. In this study, the leptin-adiponectin axis was prioritized over adiponectin isoform fractionation (high-, middle-, and low-molecular-weight), which may turn out to be key regulators of metabolic status in the mother-fetal axis. Finally, the lack of longitudinal infant follow-up prevented evaluation of whether early adipokine patterns predict postnatal growth or metabolic outcomes.

## 5 Conclusion

This study demonstrates that leptin and adiponectin, along with indices such as the FLI and LAR, might be possible indicators metabolic dysregulation associated with GDM and altered fetal growth.

Elevated leptin, LAR, and FLI in maternal plasma characterize a hormonal milieu of insulin resistance, low−grade inflammation, and adipose tissue dysfunction. These alterations impair placental nutrient allocation, linking metabolic stress to fetal adiposity, small birth weight, and metabolic programming. Maternal dysfunction exerts a stronger influence on leptin dynamics than cord blood profiles.

Higher cord adiponectin in diet-treated GDM vs. non-GDM suggests compensatory upregulation to counter mild hyperglycemia, enhancing insulin sensitivity and anti-inflammation, but may accelerate early postnatal weight gain.

Adipokine profiling identifies metabolic disturbances of pregnancies complicated by GDM for tailored interventions like polyphenol-rich (vegan/Mediterranean) lactation diets or exercise to restore balance. Future prospective studies incorporating complete maternal data, comprehensive metabolic profiling, and longitudinal infant follow-up are warranted to validate and expand upon our findings.

## Data Availability

The original contributions presented in the study are included in the article/**Supplementary Material**, further inquiries can be directed to the corresponding author.

## Supplementary material

Supplementary Table 1 Correlations Between Maternal/Cord Plasma Adipokines and Maternal/Neonatal Anthropometric Parameters in GDM and Non-GDM Groups.

Supplementary Figure 1 Maternal BMI impact on Plasma Leptin-Adiponectin Axis indicators in Mother-Newborn Pairs.
