# Supplementary material for: Gestational diabetes mellitus–induced adipokine dysregulation and links to metabolic programming risks
Source: Front Endocrinol (Lausanne). 2026 May 12;17:1776904. doi: 10.3389/fendo.2026.1776904 (PMC13201178; doi:10.3389/fendo.2026.1776904)
Supplement: Supplementary Table 1 — Correlations Between Maternal/Cord Plasma Adipokines and Maternal/Neonatal Anthropometric Parameters in GDM and Non-GDM Groups. [file DataSheet1.docx]

**Supplementary**

**Table S1. Correlations Between Maternal/Cord Plasma Adipokines and Maternal/Neonatal Anthropometric Parameters in GDM and Non-GDM Groups**

| Analyzed parameters | Body Mass Index | Birth Weight | Crown-heel Length | Head Circumference | Chest Circumference | Maternal Leptin | Maternal sLeptinR | Maternal FLI | Cord Leptin | Cord sLeptinR | Cord FLI |
| --- | --- | --- | --- | --- | --- | --- | --- | --- | --- | --- | --- |
| GDM group | | | | | | | | | | | |
| Body Mass Index | 1.00 |  |  |  |  |  |  |  |  |  |  |
| Birth Weight | 0.18 | 1.00 |  |  |  |  |  |  |  |  |  |
| Crown-heel Length | -0.18 | **0.75, *p*<0.0001**  ***q*<0.0001** | 1.00 |  |  |  |  |  |  |  |  |
| Head Circumference | -0.21 | **0.64, *p*<0.00003**  ***q*<0.0003** | 0.31 | 1.00 |  |  |  |  |  |  |  |
| Chest Circumference | 0.23 | **0.82, *p*<0.0001**  ***q*<0.0001** | **0.58, *p*<0.0006**  ***q*<0.005** | **0.54, *p*<0.0003**  ***q*<0.003** | 1.00 |  |  |  |  |  |  |
| Maternal Leptin | **0.61, *p*<0.002**  ***q*<0.01** | **0.51, *p*<0.007**  ***q*<0.04** | 0.24 | 0.31 | 0.38 | 1.00 |  |  |  |  |  |
| Maternal sLeptinR | -0.59 | -0.09 | 0.04 | 0.08 | -0.09 | 0.09 | 1.00 |  |  |  |  |
| Maternal FLI | **0.67, *p*<0.007**  ***q=*0.04** | 0.27 | 0.05 | -0.04 | 0.09 | **0.80, *p*<0.0003**  ***q*<0.003** | -0.11 | 1.00 |  |  |  |
| Cord Leptin | 0.07 | 0.45 | 0.52 | 0.12 | 0.52 | 0.21 | 0.07 | -0.07 | 1.00 |  |  |
| Cord sLeptinR | 0.05 | 0.16 | 0.14 | -0.04 | 0.17 | 0.07 | <0.01 | -0.01 | -0.07 | 1.00 |  |
| Cord FLI | 0.13 | 0.43 | 0.53 | 0.11 | 0.51 | 0.21 | 0.03 | -0.04 | **0.98, *p*<0.0001**  ***q*<0.0001** | -0.19 | 1.00 |
| Non-GDM group | | | | | | | | | | | |
| Body Mass Index | 1.00 |  |  |  |  |  |  |  |  |  |  |
| Birth Weight | -0.31 | 1.00 |  |  |  |  |  |  |  |  |  |
| Crown-heel Length | -0.31 | **0.59, *p*<0.009**  ***q*=0.09** | 1.00 |  |  |  |  |  |  |  |  |
| Head Circumference | -0.37 | **0.52 *p*<0.007**  ***q*=0.09** | 0.27 | 1.00 |  |  |  |  |  |  |  |
| Chest Circumference | -0.37 | **0.86,**  ***p*<0.00002**  ***q*<0.001** | 0.54 | 0.34 | 1.00 |  |  |  |  |  |  |
| Maternal Leptin | 0.25 | 0.13 | -0.19 | 0.06 | -0.13 | 1.00 |  |  |  |  |  |
| Maternal sLeptinR | 0.01 | 0.12 | 0.10 | 0.29 | 0.09 | 0.09 | 1.00 |  |  |  |  |
| Maternal FLI | **0.64 *p*<0.004**  ***q*=0.07** | -0.35 | -0.33 | -0.30 | **-0.57, *p*<0.02**  ***q*=0.18** | 0.66 | -0.13 | 1.00 |  |  |  |
| Cord Leptin | 0.23 | **0.47 *p*<0.04**  ***q*=0.29** | 0.10 | 0.47 | 0.36 | 0.13 | 0.55 | -0.10 | 1.00 |  |  |
| Cord sLeptinR | -0.03 | 0.17 | -0.09 | 0.41 | 0.16 | -0.01 | 0.11 | 0.12 | 0.24 | 1.00 |  |
| Cord FLI | 0.24 | 0.41 | 0.13 | 0.28 | 0.29 | 0.11 | 0.49 | -0.14 | **0.89, *p*<0.0001**  ***q*<0.0001** | -0.18 | 1.00 |

Spearman's rank correlation analysis was used to assess associations between variables. Spearman coefficients are presented with uncorrected *p*-values and FDR-corrected *q*-values (Benjamini-Hochberg procedure). Significance thresholds: *p* < 0.05 (uncorrected, bold) and *q* < 0.05 (FDR-corrected, bold and red). FLI, free leptin index; sLeptinR, soluble leptin receptor; non-GDM, normoglycemic group; GDM, gestational diabetes mellitus group.


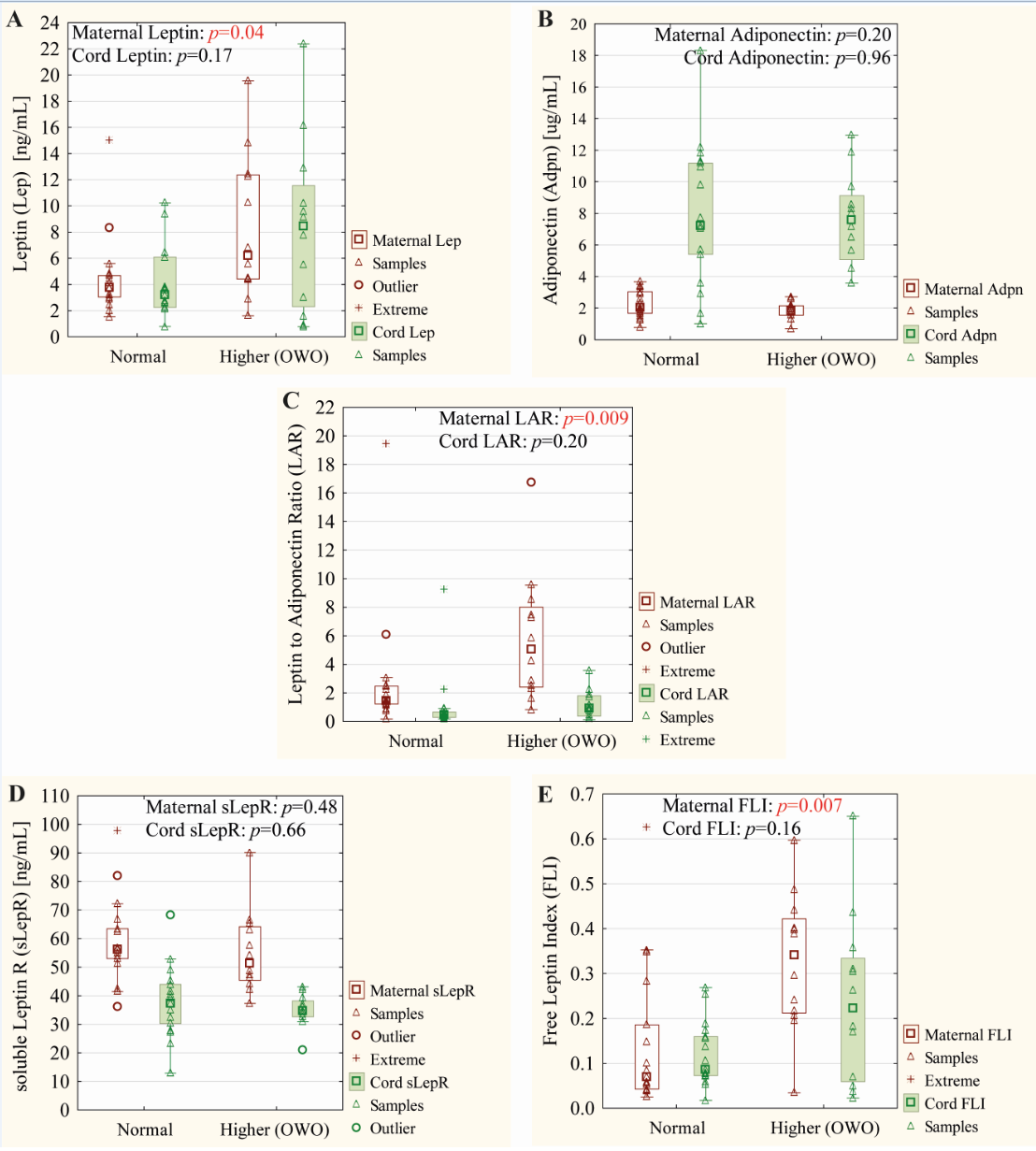


**Supplementary Figure S1. Maternal BMI impact on Plasma Leptin-Adiponectin Axis Indicators in Mother-Newborn Pairs**

Data are given as mean and median values and 25th and 75th quartiles. Kruskal–Wallis test was used for statistical calculations, and a *p*-value lower than 0.05 was regarded as significant and was marked red color.
